# AID/APOBEC cytosine deaminase induces genome-wide kataegis

**DOI:** 10.1186/1745-6150-7-47

**Published:** 2012-12-18

**Authors:** Artem G Lada, Alok Dhar, Robert J Boissy, Masayuki Hirano, Aleksandr A Rubel, Igor B Rogozin, Youri I Pavlov

**Affiliations:** 1 Eppley Institute for Research in Cancer and Allied Diseases, University of Nebraska Medical Center, Omaha, NE, USA; 2 Department of Genetics, Cell Biology and Anatomy and Munroe-Meyer Institute, University of Nebraska Medical Center, Omaha, NE, USA; 3 Department of Internal Medicine, University of Nebraska Medical Center, Omaha, NE, USA; 4 Emory Vaccine Center, Department of Pathology and Laboratory Medicine, Emory University, Atlanta, GA, USA; 5 Department of Genetics, Saint Petersburg University, Universitetskaya emb. 7/9, St. Petersburg, 199034, Russia; 6 St. Petersburg Branch of Vavilov Institute of General Genetics, St. Petersburg, Universitetskaya emb. 7/9, St Petersburg, 199034, Russia; 7 National Center for Biotechnology Information, National Library of Medicine, National Institutes of Health, Bethesda, MD, USA; 8 Institute of Cytology and Genetics, Novosibirsk, Russia

**Keywords:** APOBEC, Deaminase, Mutation, Kataegis, Cancer, Diploid yeast, Hypermutation

## Abstract

Clusters of localized hypermutation in human breast cancer genomes, named “kataegis” (from the Greek for thunderstorm), are hypothesized to result from multiple cytosine deaminations catalyzed by AID/APOBEC proteins. However, a direct link between APOBECs and kataegis is still lacking. We have sequenced the genomes of yeast mutants induced in diploids by expression of the gene for PmCDA1, a hypermutagenic deaminase from sea lamprey. Analysis of the distribution of 5,138 induced mutations revealed localized clusters very similar to those found in tumors. Our data provide evidence that unleashed cytosine deaminase activity is an evolutionary conserved, prominent source of genome-wide kataegis events.

**Reviewers:**

This article was reviewed by: Professor Sandor Pongor, Professor Shamil R. Sunyaev, and Dr Vladimir Kuznetsov.

## Findings

Sequencing of cancer genomes has revealed that tumors contain numerous mutations [1]. It is unclear how such a large number of DNA sequence changes are induced and what factors influence the distributions of the rates of mutations among cells and within regions of the genome during tumor development. Recently, regions of localized hypermutation, called kataegis, have been detected in breast cancer genomes [2]. Based on the prevalence of C:G->T:A transitions in these regions and the sequence context of the mutations, it has been hypothesized that mutation clusters in cancer are induced by AID/APOBEC editing deaminases [2,3]. This is consistent with the ability of deaminases to produce multiple deaminations in ssDNA *in vitro* that can be recovered as clustered mutations in bacteria [4,5]. Using Sanger sequencing, Liu *et al.* discovered AID-induced mutations in various loci in B-cells [6]. APOBEC3B can introduce base substitutions (detected by 3D-PCR) in a reporter gene integrated into the genome of a human cell line [7]. However, a direct link between APOBECs and kataegistic clustered mutations has not been reported. A yeast model is an efficient approach to study this phenomenon. Regions of ssDNA are recognized as a prerequisite for kataegis-like events induced by an alkylation agent in yeast, and by extrapolation, have been proposed to be a prerequisite for the kataegistic action of deaminases in humans [3]. Double-strand DNA breaks in the vicinity of a reporter gene synergistically stimulate mutagenesis by AID, and in yeast this behavior might be related to the generation of ssDNA during homologous recombination [8].

We examined kataegis induced in diploid yeast by the most mutagenic AID/APOBEC protein, PmCDA1 from sea lamprey [9]. AID/APOBECs belong to a superfamily of proteins with diverse functions, from RNA editing to humoral and innate immunity and DNA demethylation [10]. Intriguingly, the basis of such a plethora of functions is a relatively simple reaction: the deamination of cytosine to uracil in ssDNA or RNA. During replication, uracil pairs with adenine resulting in a C:G->T:A transitions in the next round of replication. We expressed an exogenous *PmCDA1* gene in a diploid yeast strain LAN210 defective for uracil-DNA-glycosylase (*ung1*). The Ung1 protein excises uracil during base excision repair in yeast; therefore, *UNG1* inactivation sensitizes yeast to APOBEC effects. At the same time, Ung1 deficiency abrogates PmCDA1’s ability to induce mitotic recombination [9]. PmCDA1-induced canavanine-resistant (Can^r^) mutants were selected and their genomes were resequenced using the Illumina platform, which involved the mapping of reads corresponding to the mutant clones against a reference produced by DNA sequencing and *de novo* assembly of the basic LAN210 genome. We also sequenced the genomes of Can^r^ mutants induced in an isogenic diploid strain by the powerful base analog mutagen 6-hydroxylaminopurine (HAP), one that also (like PmCDA1 in the *ung1* strains) is not excised by base excision repair and does not induce recombination in yeast ([11,12] and references therein). Therefore, the distributions of mutations obtained in our study represent unbiased snapshots of genome-wide mutagen-induced base substitutions.

**Figure 1:**
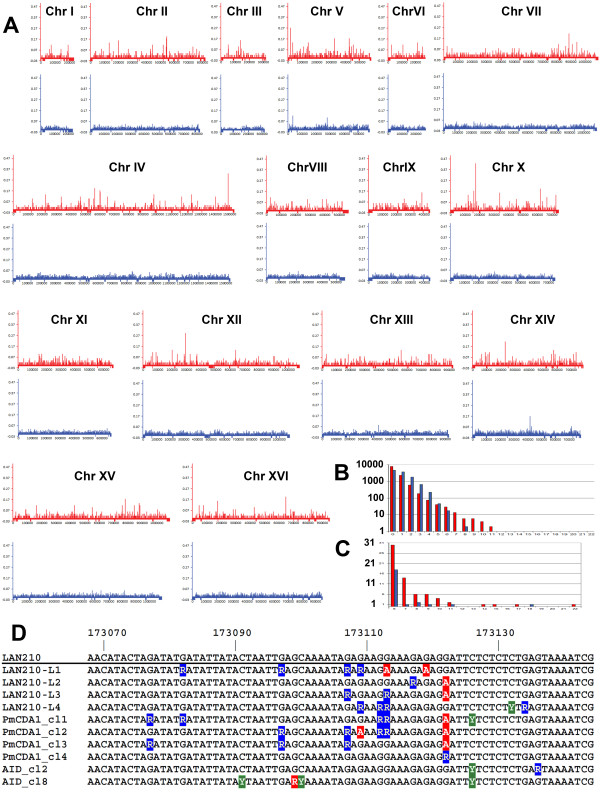
**Improbable hotspots of mutagenesis induced by PmCDA1 in yeast genomes. A**. Genome-wide distributions of mutation (also called single nucleotide variants, SNVs) frequencies in PmCDA1 (red) and HAP (blue) induced mutants. SNV frequencies across the entire genome are shown. Negative values (i.e., bars below the X axis) indicate regions of the genome excluded from analysis (mostly repetitive regions). **B**. Distribution of 1 Kb windows with the indicated number of SNVs for PmCDA1 (red bars) and HAP (blue bars), log scale. **C**. Same as **B**, but using a linear scale, and where values >5 are shown. **D**. Multiple alignment of the DNA sequences of the hypermutable region on chromosome X from different mutant clones: LAN210-L1 – LAN210-L2 – clones used for genomic sequencing; PmCDA1-cl1 – PmCDA1-cl4 – independent mutants with this region sequenced by the Sanger method; AID_cl2 and AID_cl8 – AID-induced mutants that were found to contain mutations in this region. Homozygous G->A substitutions are red; heterozygous G->A substitutions are blue Rs; heterozygous C->T substitutions are green Ys.

To analyze the distribution of mutations in resequenced genomes, we pooled the results from four genomes of PmCDA1-induced mutants and eight genomes of HAP-induced mutants. Each chromosome sequence was divided into 1-Kbp intervals, and the number of mutations per window was calculated. The mutation densities were calculated across the entire genome and plotted as a function of each interval’s chromosomal coordinate for all 16 chromosomes (Figure 1A). The distributions of the intervals with a given number of mutations are shown as insets in Figure 1B and C. Mutation randomness analysis was done using C.A.MAN [13,14] by calculating the threshold values of the mutation densities per window. The details of experimental procedures are in Additional File 1 and in the article by AGL, Elena G. Stepchenkova, Irina S.-R. Waisertreiger, Vladimir N. Noskov, AD, James D. Eudy, RJB, MH, IBR and YIP, which is currently under review. Analysis of the distribution of HAP-induced mutations revealed three classes of windows. The first class includes windows with 5 or less mutations, the second class includes highly mutable regions (6 to 18 mutations). The threshold value of six mutations per window was chosen as for determining highly mutable windows. Analysis of the PmCDA1-induced mutations also revealed three classes of windows. The first class includes windows with 4 or less mutations, the second class includes highly mutable windows with 5 to 11 mutations per window, and the third class comprises obvious hypermutable windows (number of mutations 14, 15, 17, and 22). The threshold value of five mutations per window was chosen for determining highly mutable windows. Thus, for the respective classes of mutagenic agents, the thresholds for highly mutable 1-Kb intervals were defined as those that contained six or more HAP-induced mutations or five or more PmCDA1-induced mutations.

Overall, we found that mutation densities ≥ 5 and ≥ 6 mutations per Kb (~0.078% for PmCDA1 and 0.046% for HAP, respectively) indicate a non-random mutation induction. We found 108 such regions for PmCDA1 and 24 for HAP (see Figure 1B, C and Table 1 for the distributions of the actual counts of mutations per Kb).

**Table 1:** Distributions of mutations in 1000 bp windows

**Number of mutations**	**Number of windows**	
	**HAP-induced**	**PmCDA1-induced**
0	4683	7884
1	3705	2334
2	1842	613
3	674	188
4	226	76
5	**47**	42
6	**18**	**30**
7	**1**	**14**
8	**2**	**6**
9	**1**	**6**
10	0	**4**
11	**1**	**2**
12	0	0
13	0	0
14	0	**1**
15	0	**1**
16	0	0
17	0	**1**
18	**1**	0
19	0	0
20	0	0
21	0	0
	0	**1**

Highly mutable regions are shown in bold and underlined.

These numbers underscore the striking differences between the genome-wide distributions of PmCDA1- and HAP-induced mutations in the sequenced clones (Figure 1). Deaminase-induced mutation clusters are evident at various genomic loci (Figure 1A, red graphs), whereas the density of HAP-induced mutations is much more uniform (Figure 1A, blue graphs; see also Figure 1B and C for mutation distributions). Very dense clusters of deaminase-induced mutations were found on chromosomes IV, V, VII, X, XII, XIV and XVI (Figure 1A). The most striking example of a kataegistic deamination cluster is on chromosome X (at chromosomal coordinate ~174000, between the *URA2* and *TRK1* genes, i.e., the highest peak in chromosome X in Figure 1A). In this region, we observed 22 mutations in a 1,000 bp interval, which corresponds to ~0.43% of the mutations found in four genomes (yeast haploid genome size is ~12 Mb). All four sequenced clones possess mutations in this region (Figure 1D). These results were confirmed by Sanger sequencing. We PCR-amplified ~2,100 bp between the *URA2* and *TRK1* genes and sequenced the resulting product using three primers (primer sequences are available upon request). In contrast, mutation clusters were practically not found in the genomes of HAP-induced mutants. Sequencing of the region between *URA2* and *TRK1* in four independent PmCDA1-induced mutants confirmed that this genomic region is highly prone to dense mutation clusters caused by localized “thunderstorms” of enzymatic deamination. At position 173,122 on chromosome X, a G->A transition was found in 6 out of 8 clones, and this mutation was homozygous in 5 clones (Figure 1D). Given the average mutation load in mutant clones and the GC content of the yeast genome, the probability of observing this by chance is ~9x10^-43^, assuming independent mutations on homologous chromosomes. We also sequenced this hypermutable region in Can^r^ mutants induced by a different deaminase, human AID, and found mutations in 2 out of 12 clones (Figure 1D). Since this AID is less active than PmCDA1 in yeast, we concluded that hypermutability of this region is independent of the deaminase studied, i.e., it has certain fundamental properties making it prone to mutation induction by these enzymes. In addition, a strong strand bias was found in this region (predominantly G->A mutations), which may be a result of preferential deamination on the non-transcribed strand of nearby genes.

The clusters of cytosine deaminase-induced mutations described here are very similar to those found in cancer cells [2,3]. They can be explained by the processive activity of AID/APOBEC enzymes [4] in genomic regions where ssDNA is exposed and is highly accessible to the deaminase. The level of adjacent transcription is one of the factors that can influence the accessibility of genomic DNA to deaminases. Our results establish, for the first time, a direct link between cytosine deaminases and kataegis. It should be noted that in our system the induction of homologous recombination was suppressed, either by the nature of the mutagen (HAP) or the use of *ung1* strains for PmCDA1. This makes our model more similar to human cells where this type of recombination is relatively rare. The use of diploid strains was also critical for uncovering the full mutagenic potential of the deaminase. In terms of inter-mutation distances, the strongest mutation clusters observed in our study (such as the one shown on Figure 1D) resemble the micro-clusters found in the macro-clusters in the kataegistic regions of breast cancer genomes (See Figure 4A in [2]). We found that PmCDA1 predominantly introduces mutations in ATC motifs (more details in the manuscript under review, see above). Similarly, a group of mutations in breast cancer kataegis is characterized by a TCX motif signature. Further studies will reveal if PmCDA1, a lamprey enzyme, possesses a conserved specificity. Importantly, our study reveals that the AID/APOBEC proteins can induce kataegis in the genome. The nature of the deaminase participating in this process will dictate the sequence motifs where mutations occur.

These findings have broad implications for cancer biology and evolution, especially in the context of the recent discovery of AID/APOBEC-like proteins in various prokaryotes and eukaryotes [15]. Thus, kataegis might be a widespread phenomenon in the evolution of different forms of life. Recently, localized mutation clusters were discovered in yeast mutants obtained by a different mutagen, methyl metanesulfonate (MMS) [3]. At present it is not possible to predict the impact of kataegis on the existing evolutionary models of mutation frequencies, which assume the independence of mutation events. Kataegis may also significantly influence the outcome of other studies of discrete evolutionary events, e.g. the detection of recombination patches.

## Abbreviations

UNG: Uracil DNA glycosylase (uracil-*N*-glycosylase); HAP: 6-hydroxylaminopurine; SNV: Single nucleotide variant; ssDNA: Single-stranded DNA.

## Competing interests

The authors declare no competing interests.

## Authors’ contributions

AGL performed the experiments, analyzed the data and wrote the manuscript. AD performed the NGS sequencing. RJB participated in data analysis and in the writing of the manuscript. MH performed the cloning and characterization of PmCDA1. AAR participated in confirmatory Sanger sequencing. IBR participated in the data analysis and writing. YIP conceived the work, discussed its experimental aspects and the outline of the work and finished writing the manuscript. All authors edited and approved the final version.

## Reviewers’ comments

Reviewer's report Title: AID/APOBEC cytosine deaminase induces genome-wide mutation clusters Version: 1 Date: 1 November 2012 Reviewer number: 1 Professor Sandor Pongor

Report form:

Regions of localized hypermutations – so-called kataegis regions – were recently found to be colocalised with regions of somatic genome rearrangements in cancer genomes. As C:G->T:A transitions were overabundant in these regions, it was hypothesized that AID/APOBEC editing deaminases that are responsible for cytosine to uracil deamination in single-stranded DNA or RNA, may be one of the causative agents generating localized hypermutations. However plausible in the chemical sense, this hypothesis is difficult to prove by experiment. In this Discovery Note, Lada *et al.* describe an experiment designed to provide a very interesting piece of supporting evidence to this hypothesis. The authors used a diploid yeast sensitized to deamination effects by the removal of the uracil DNA glycosylase gene (ung1) as the model organism. Then they expressed a hyperactive AID/APOBEC protein from sea lamprey in this organism and explored the distribution of mutations along the chromosomes. It was found that distribution of these mutations is highly uneven, and the differeces are especially striking in comparison with those induced by the base analog mutagen 6-hydroxlaminopurine (HAP) which was used as a control. The findings are straightforward and provide strong support to the hypothesis that unleashed AID/APOBEC may be the causative agents of hypermutations found in cancer genomes. The link between the two phenomena is that clusters of deaminase-induced mutations in yeast are very similar to those found in cancer cells. It would be very interesting to see a more detailed description of this similarity. Are there similarities in the sequence contexts? It is a convincing argument that HAP-induced mutations in ung1- mutants are entirely random, but perhaps there are other examples of or analogies with more uneven mutations in the literature where, for instance, the context of the mutations are different.

Author’s response: *We are glad that Dr. Pongor considered our study interesting and convincing. We would like to thank Dr. Pongor for the constructive suggestions on manuscript improvements. We have discussed the sequence context of the mutations found in our study and in breast cancer samples, as well as the recent paper (Roberts et al.) where clustered mutations were induced in yeast by a different mutagen*-*MMS.*

The experiments are complex, and the details are described in an experiment under review. I would suggest the authors add a brief description of the experiment as an appendix to this note.

Author’s response: *As suggested, we have added a short overview of experiments undertaken. We also provide more details in the responses to Reviewer #3. In addition, since the format of the Discovery Notes is adapted to the short communications, we refer the interested readers to the paper (Lada et al.) that is currently under review. This paper contains the details of experiments undertaken.*

In summary, I find the experiment well-designed and the conclusions convincing.

Reviewer’s response: *I accept the revisions*.

Quality of written English: Acceptable

Reviewer's report Title: AID/APOBEC cytosine deaminase induces genome-wide mutation clusters Version: 1 Date: 15 November 2012 Reviewer number: 2 Professor Shamil R. Sunyaev

Report form:

I find this manuscript to be of great interest. Two recent publications reported presence of mutation clusters induced by APOBEC proteins in cancer genomes, shedding new light on the nature of spontaneous somatic mutagenesis. This manuscript provides experimental evidence supporting the hypothesis of recent observational studies. The authors report that genomes of yeast mutants carrying the hypermutagenic deaminase contained mutation clusters highly similar to clusters (putatively caused by APOBECs) observed in tumor genomes. This is an important result and I have no suggestions for improvements.

Author’s response: *We are excited that Professor Sunyaev found our work to be of great interest and that it provides new information on spontaneous mutagenesis.*

Reviewer’s response: *I did not have any concerns with the manuscript.*

Quality of written English: Acceptable

Reviewer's reportTitle: AID/APOBEC cytosine deaminase induces genome-wide mutation clustersVersion: 1Date: 15 November 2012Reviewer number: 3Dr Vladimir Kuznetsov

Report form: Comments

Recent papers (2,3) have provided detail descriptions of clustered mutation sites in the genomes of four human cancers and in yeast cells. In (3) functional and structural association of APOBEC proteins with clustered mutation have been suggested. However, more direct functional associations of APOBEC family member(s) proteins with clustered mutations have to be carrying out. In this study, the authors used sequencing technique and their yeast model to study genome clusters of hypermutation activity of deaminases PmCDA1 and AID.

Major concerns and my recommendations:

1. Analysis of the literature is essentially incomplete

The authors claimed: “… a direct link between APOBEC deaminase activity and genome-wide hypermutagenesis is still lacking.” However, this claim has to be debated. Atomic force microscopy studies provided direct evidence of the structural details of direct interaction of APOBEC3G with ssDNA on a specific site at a sing molecular level and at nanometer resolution (Shlyakhtenko *et al.*, 2011). Yamane *et al.* (2011) reported about deep sequencing analysis of mutations and identification of the genomic targets of AID in mouse B-cells and provided the evidences of association of ssDNA hypermutation sites with APOBEC binding motif. At least two papers reported functional and structural connection between AID/APOBECs and genome-wide hypermutation (Klein *et al.* 2011; Yamane *et al.* 2011). Both papers studied the impact of AID in mouse B-cells at the genome scale.

Author’s response to 1: *We thank Dr. Kuznetsov for extensive review of our paper that took significant effort and almost two months. In response to the critique we added a more balanced discussion of the papers that we deemed ultimately related to our study (references 2–8). As to papers mentioned by the reviewer, a very interesting article by Yamane et al. (2011) is devoted to the construction of ChIP-based whole-genome maps of AID and RPA occupancies and is neither analyzing genome-wide mutation distributions nor report the discovery of clustered mutations. Moreover, in our opinion, interpretation of the very solid experimental results of this study should be re-considered, because they are in direct disagreement to data obtained in our lab (Lada et al., 2011) and by Dr. Myron Goodman’s group (Pham et al., 2008; Chelico et al., 2009). The paper by Klein et al. (2011) presents a very thorough genome-wide study where the authors used a powerful translocation-capture sequencing method to map chromosomal rearrangements in B lymphocytes. Although the authors do report that translocation hotspots were accompanied by the base substitutions, we would like to point out that, similar to the paper by Yamane et al. (2011), the genome-wide mutagenesis study is not performed in this study and mutational clusters are not detected. Moreover, the paper by Nik-Zainal (2012) reporting the discovery of kataegis and discussing the potential involvement of the APOBEC protein in the formation of clusters of mutations was published later than all of the mentioned papers. In addition, studies of activated B-cells, which provide the natural environment for the AID activity, do not explain how the genomes of breast cells become edited by the APOBEC proteins.*

2. There is no description of the sequencing methods. Even the number of reads was not reported.

3. Raw and processed data are not available.

4. Sequencing generation, sequence data analysis, genome assembly and mapping procedures and results of these steps omitted.

Author’s response to 2–4: *The format of the Discovery Notes does not allow us to include all the Materials and Methods related to our data. We refer to our parallel paper (Lada et al., currently under review) where all the details of experimental procedures and data analysis are described in detail. However, we have added a short description of materials and methods used in this manuscript, including the numbers of reads, coverage and the NCBI accession number for the raw data. This text is available as an Additional File**1*.

5. Authors did not provide systematic evidences of accuracy of their finding.

Statistical model(s) of background noise, testing methods, and analysis of experimental results are not reported. There are no any estimates of specificity and sensitivity of the proposed experimentally detected mutation sites and clustered mutations associated with PmCDA1 and AID activity.

Author’s response to 5: *All draft reference genome assemblies performed in this study were manually edited and assembly errors were excluded from analysis. The remaining questionable few regions were sequenced using the Sanger method to confirm or reject the SNVs detected. The detailed description of these procedures is beyond the scope of the Discovery Notes, see response to comments 2–4.*

6. A work needs to develop an analysis of the boundaries of clustered mutations; result should include the frequency tables of all observed mutation transitions occurred in clustered mutation as well as in the regions out of the clusters.

Author’s response to 6: *There are methods to analyze the clustering of mutations that attempt to locate the boundaries of the regions with an elevated frequency of mutations (P.J. Gearhart, D.F. Bogenhagen, 1983. Clusters of point mutations are found exclusively around rearranged antibody variable genes. Proc. Natl. Acad. Sci. U.S.A. 80, 3439–3443; H. Tang, R.C. Lewontin 1999. Locating regions of differential variability in DNA and protein sequences. Genetics 153, 485–495). These methods, however, require a much higher frequency of mutations per nucleotide and were tested for relatively short sequences.*

*We have used a classification approach to analyze the distribution of mutations across yeast chromosomes using non-overlapping windows. This method is not capable of finding the exact boundaries of hypermutable regions, however it allows for the detection of the general trends in a robust way. It is described in more details in the revised draft and in new Additional File**1*.

7. There are no final lists of clustered mutations and their genome coordinates and biological interpretation.

Author’s response to 7: *We have added Table 1, which contains distribution of mutations in 1 Kb windows.*

8–9. The number of C to T substitution mutation is reported only for two genes on chrX; There are no quantitative data and numerical/statistical characteristics for clustered mutation sites, any other genes, regions and chromosomes.9. Statistical distributions of all base transitions (e.g. % of C to T, G to A etc.) should be presented and discussed. The work should provide mutation’ classification and include description of the substitution mutation in the clusters on positive and negative strands and supporting by APOBEX/AID motif(s) co-localization.

Author’s response to 8–9: *See response to comments 2–4.*

10. There is no comparison of the results of this genome-wide finding with alternative studies.

Author’s response to 10: *We have included a more extensive discussion of the results by Roberts et al. (2012).*

11. A reason of using 6-hydroxlaminopurine (HAP) treated cells as a negative control should be explained.

Author’s response to 11: *We are especially grateful for this comment. One of the major emphases of the paper is to study mutagenesis in diploid yeast independent of recombination, which is uniquely frequent in this organism. We have chosen conditions and mutagens when induced recombination is suppressed and the situation is closer to processes in human cells (both HAP and PmCDA1 in ung1- strains does not induce recombination in yeast). We have updated the text accordingly to make this more transparent.*

Summary:

This work is essentially incomplete and poorly performed; there is no way to reproduce its methods, results and evaluate their actual value.

Author’s response: *See answers to comments 2–4 and 8–9. We also think that even without the knowledge of fine experimental details there is a straightforward way to reproduce the results of this work by expression of PmCDA1 gene in diploid ung1- yeast strain or treatment by HAP, selection of mutants and genome sequencing.*

Quality of written English: Not suitable for publication unless extensively edited

Author’s response: *Please see evaluation by reviewers 1 and 2. Nevertheless, we have put forth additional effort and we have carefully edited the manuscript.*

Reviewer’s response: 

1. “….*clusters very similar to those found in tumors*”.

What kind of parameter(s) is similar? What kind of similarity/dissimilarity measure(s) between mutation clusters in yeast and human cancer genomes was used? Is there some statistical estimation? If it is statistical-based analysis, the test and confidence values should be reported.

2. “*We also think that even without the knowledge of fine experimental details there is a straightforward way to reproduce the results of this work by expression of PmCDA1 gene in diploid ung1- yeast strain or treatment by HAP, selection of mutants and genome sequencing*.”

Unfortunately, NGS is not well matured and standardized technic, specifically, in context of ‘The details’ of experimental procedures, data analysis and interpretation. Perhaps many readers of BD whose have an experience to use NGS technics and corresponding analytical method, should disagree with the authors point. Specifically -processing, alignment, mapping results and analysis of data are not trivial steps and are usually reported in publications as regular (not referred to unpublished data). As usual publication practices, it should be presented in suppl. file.

3. “*primer sequences are available upon request*”.

Why? This information should be present in the work, if no commercial interest.

4. “*Importantly, our study reveals that the AID/APOBEC proteins can induce kataegis in the genome*”

This conclusion should be too strong. The inducer(s) of “kataegis” were not defined; it might be identified in future works.

5. “*Our data provide evidence that unleashed cytosine deaminase activity is an evolutionary conserved, prominent source of genome-wide kataegis events*.”

It might be too strong conclusion. The evidences of the evolution conservation of cytosine deaminase activity in “genome-wide kataegis” loci across species were not reported and they should be done for specific kataegis loci if any.

6. Minor: *NGS Instrument model should be indicated in the manuscripts*.

Quality of written English: Acceptable.

## Supplementary Material

Additional file 1 Supplementary Experimental Procedures. Click here for file
