# Supplementary material for: AID/APOBEC cytosine deaminase induces genome-wide kataegis
Source: Biol Direct. 2012 Dec 18;7:47. doi: 10.1186/1745-6150-7-47 (PMC3542020; doi:10.1186/1745-6150-7-47)
Supplement: Additional file 1 — Supplementary Experimental Procedures. [file 1745-6150-7-47-S1.docx]

**Supplementary Experimental Procedures.**

**Genome sequencing.** We obtained about 40 million 2x101-bp paired-end reads for each genome. Reads from the un-mutagenized LAN210 strain were used to produce a high-quality DNA sequence assembly for an “isogenic reference” genome, with an average coverage depth of 300x. For the mutant strains, mapping of their reads against this “isogenic reference” genome assembly showed that both agents induce about a thousand mutations per diploid genome, ten-fold more than in isogenic haploid genomes. Details of our next-generation whole-genome DNA sequencing, construction of the reference genome of our basic strain by *de novo* assembly from raw reads (NCBI Sequence Read Archive, [www.ncbi.nlm.nih.gov/sra](http://www.ncbi.nlm.nih.gov/sra), [SRA: SRA057025]), and the reference assembly of reads obtained by sequencing of genomes of mutants and single-nucleotide variant (SNV) detection are described in an article by AGL, Elena G. Stepchenkova, Irina S.-R. Waisertreiger, Vladimir N. Noskov, AD, James D. Eudy, RJB, MH, IBR, YIP, which is currently under review.

**Statistical analysis of mutation distributions.** Mutation randomness analysis was done using C.A.MAN [[1](#_ENREF_1)] by calculating the threshold values of the mutation densities per window. Briefly, this program classifies each window according to different mutation probabilities in the window, and each window should belong to only one class. The distribution of mutation number per window in each class is approximated by the Poisson distribution and an overall distribution is regarded as a mixture of Poisson distributions. Variations in mutation frequencies among windows of the same class are assumed to be due to random reasons (since mutation probability is the same for all sites in one class), whereas differences between mutation frequencies among windows from different classes are statistically significant. The C.A.MAN classification procedure that separates the distribution into classes is iterative and each iteration includes maximization and estimation procedures similar to the methods used for the detection of mutation hotspots (reviewed in [[2](#_ENREF_2)]). Analysis of the distribution of HAP-induced mutations revealed three classes of windows. The first class includes windows with a number of mutations less than or equal to 5; and the second class includes highly mutable regions with the mutation frequency from 6 to 18. The threshold value of six mutations per window was chosen for determining highly mutable windows. Analysis of the number of PmCDA1-induced mutations revealed three classes of windows. The first class includes windows with a number of mutations less than or equal to 4; the second class includes highly mutable windows with the mutation frequency from 5 to 11; and the third class comprises obvious hypermutable windows (number of mutations 14, 15, 17, and 22). A number of five mutations per window was chosen as the threshold value for determining highly mutable windows.

**References:**

1. Bohning D, Dietz E, Schlattmann P: **Recent developments in computer-assisted analysis of mixtures.** *Biometrics* 1998, **54:**525-536.

2. Rogozin IB, Pavlov YI: **Theoretical analysis of mutation hotspots and their DNA sequence context specificity.** *Mutat Res* 2003, **544:**65-85.
